# Betulinic Acid-Brosimine B Hybrid Compound Has a Synergistic Effect with Imatinib in Chronic Myeloid Leukemia Cell Line, Modulating Apoptosis and Autophagy

**DOI:** 10.3390/ph16040586

**Published:** 2023-04-13

**Authors:** Julia Biz Willig, Nádia Miléo Garcês de Couto, Débora Renz Barreto Vianna, Camila da Silveira Mariot, Simone Cristina Baggio Gnoatto, Andréia Buffon, Diogo André Pilger

**Affiliations:** 1 Post-Graduation of Pharmaceutical Science Program, Faculty of Farmacy, Federal University of Rio Grande do Sul, Porto Alegre 90610-000, Brazil; 2 Laboratory Biochemical and Cytological Analysis, Federal University of Rio Grande do Sul, Porto Alegre 90610-000, Brazil; 3 Laboratory of Phytochemistry and Organic Synthesis, Federal University of Rio Grande do Sul, Porto Alegre 90610-000, Brazil

**Keywords:** betulinic acid, brosimine B, imatinib, synergism, K-562, chronic myeloid leukemia

## Abstract

Chronic myeloid leukemia (CML) is a myeloproliferative disease characterized by the formation of the BCR-ABL (breakpoint cluster region-Abelson) oncoprotein. As many patients display therapeutic resistance, the development of new drugs based on semisynthetic products represents a new potential therapeutic approach for treating the disease. In this study, we investigated the cytotoxic activity, possible mechanism of action of a hybrid compound of betulinic acid (BA) and brosimine B in CML cell lines that are sensitive (K-562) and resistant (K-562R) to imatinib, in addition to evaluating lower doses of imatinib in combination with the hybrid compound. The effects of the compound, and its combination with imatinib, on apoptosis, cell cycle, autophagy and oxidative stress were determined. The compound was cytotoxic in K-562 (23.57 ± 2.87 μM) and K-562R (25.80 ± 3.21 μM) cells, and a synergistic effect was observed when it was associated with imatinib. Apoptosis was mediated by the caspase 3 and 9 intrinsic pathway, and cell cycle evaluation showed arrest at G0/G1. In addition, the hybrid compound increased the production of reactive oxygen species and induced autophagy by increasing LC3II and Beclin-1 mRNA levels. Results suggest that this hybrid compound causes the death of both imatinib-sensitive and -resistant cell lines and may hold potential as a new anticancer treatment against CML.

## 1. Introduction

Chronic myeloid leukemia (CML) is a myeloproliferative neoplasm characterized by clonal expansion of pluripotent hematopoietic stem cells leading to the accumulation of myeloid cells in the bone marrow and peripheral blood [1]. The disease, in most cases, is characterized by the presence of a specific cytogenetic abnormality, the Philadelphia chromosome (Ph+), resulting from the translocation t(9; 22) (q34; q11) between the ABL oncogene on chromosome 9 and the BCR gene on chromosome 22. Inhibitors of tyrosine kinase (TKI) are the main form of treatment for CML [2]. Imatinib, the first line of treatment for CML, inhibits BCR-ABL (breakpoint cluster region-Abelson) tyrosine kinase activity by blocking the ATP-binding site [2]. However, many patients demonstrate therapeutic resistance after some period of use or do not respond to treatment, even with the second and third generation of TKIs. Currently, the search for new drugs based on natural or semisynthetic products has gained prominence in the development of new therapeutic options for hematological tumors [3].

Terpenes, polyphenols and alkaloids are secondary metabolites extracted from natural products, and several studies in the literature have reported on their antitumor activity [4]. Flavonoids, a subtype of polyphenols, modulate signaling pathways involved in cell survival and proliferation, triggering apoptosis and reducing immune responses and cascades of inflammation [5]. Flavan brosimine B, found in *Brosimum acutifolium* barks, has anti-inflammatory and anti-rheumatic activity [6] and, in the lymphoma cell line, has demonstrated cytotoxic activity in vincristine-sensitive and -resistant cells [7].

Betulinic acid (BA) is a pentacyclic triterpene found in many plants, fruits and vegetables; this molecule has several therapeutic activities and is demonstrated to be anti-inflammatory, antiviral, antibacterial, antimalarial, immunomodulatory, antidepressive and antitumor agent [8,9,10]. The antitumor activity of BA has been described in several tumor lines, such as melanoma, neuroblastoma, glioblastoma, colon, hepatocellular, lung, prostate, breast, ovary, cervical carcinoma and leukemia [11,12]. Its main mechanism of action is the induction of cell death by apoptosis, autophagy, modulation of kinase proteins and inhibition of DNA topoisomerases, among others [10,11].

Currently, several strategies are being used to try to reduce the therapeutic resistance to chemotherapy. One of these strategies is to associate new molecules with drugs that are already commercially available, and another is to search for molecules capable of promoting different cell death mechanisms in order to achieve maximum effectiveness, reducing doses and the chance of acquiring resistance [13,14]. Several studies have evaluated the cytotoxic activity of hybrid compounds. Triazole-substituted quinazoline derivatives have been synthesized for their epidermal growth factor receptor (EGFR) tyrosine kinase activity and demonstrated moderate activity in tumor cell lines (HCT116, MCF-7 and PC-3) [15]. BA was hybridized with a synthetic artemisinin derivative and exhibits tumor cell activity in glioma, astrocytes and neurons [16]. In CML cells, hybrid compounds containing part of the structure of imatinib, nilotinib and dasatinib have been developed to enhance BCR/ABL inhibition activity [17]. In this study, we investigated the cytotoxic activity and possible mechanisms of action of a hybrid compound of BA and brosimine B in CML BCR-ABL + cell lines that are sensitive (K-562) and resistant (K-562R) to imatinib. In addition, we evaluated lower doses the combination of imatinib with this hybrid compound in K-562 cells.

## 2. Results

### 2.1. The Hybrid Compound Inhibits the Growth of Human Leukemia Cells

**Figure 1:**
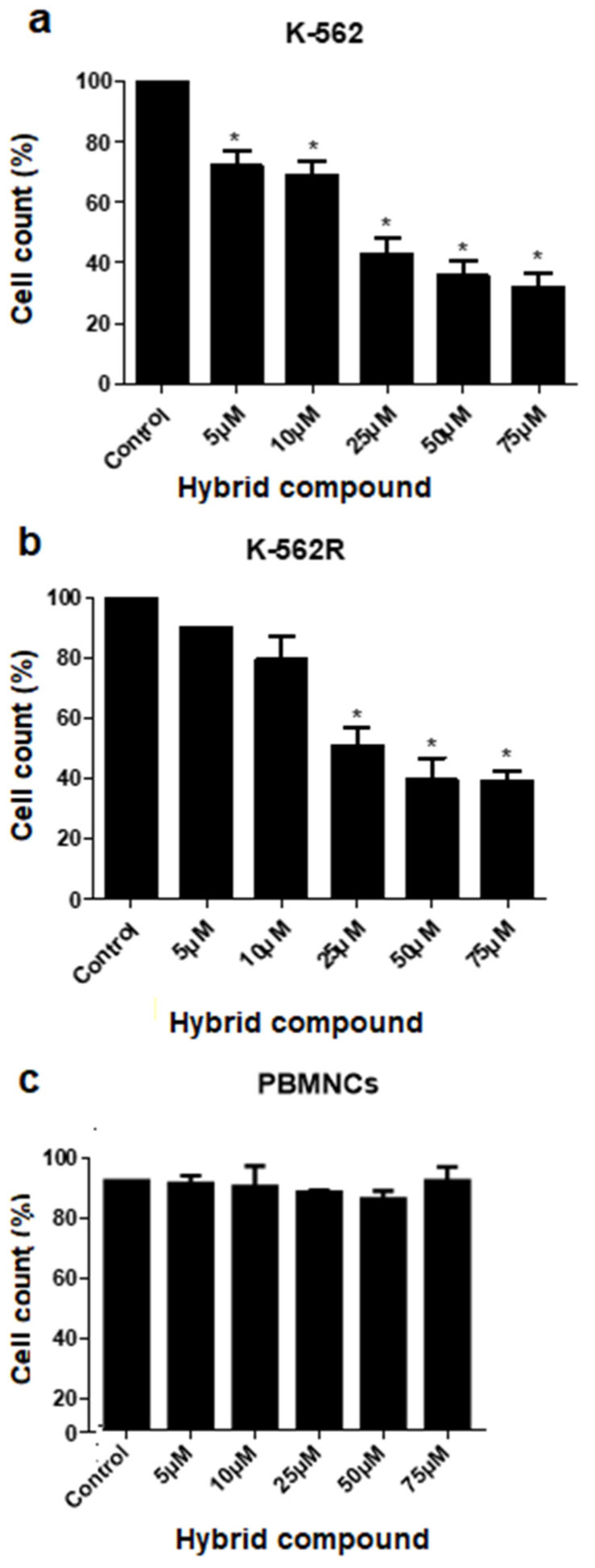
Inhibition of growth after 48 h treatment with different concentrations of the hybrid compound in (**a**) K-562 cells, (**b**) imatinib-resistant K-562 cells (K-562R) and (**c**) peripheral blood mononuclear cells (PBMNCs). Data are reported as mean ± standard deviation (SD) of three experiments (* *p* < 0.05; relative to untreated control cells).

Cell counting was used to study the cytotoxic effects of the hybrid compound on the K-562 and K-562R cell lines. As shown in Figure 1a,b, the hybrid compound induced a cytotoxic effect on K-562 and K-562R cells, where the IC_50_ values were 23.57 ± 2.87 μM and 25.80 ± 3.21 μM, respectively. The same analysis in peripheral blood mononuclear cells (PBMNCs) showed that the hybrid compound did not have a cytotoxic effect on these control cells (Figure 1c). The SI values, obtained from the ratio of EC_50_ values of the compound in PBMNCs cells and the IC_50_ values in the cancer cell lines, were 11.06 and 10.11 for K-562 and for K-562R cells, respectively.

### 2.2. The Combination of the Hybrid Compound and Imatinib Synergistically Inhibited the Growth of Human Leukemia Cells

**Figure 2:**
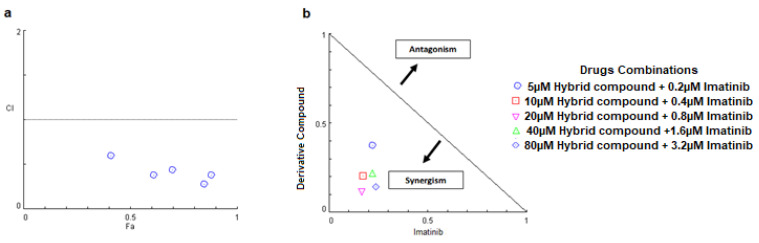
The combination of the hybrid compound with imatinib synergistically inhibited the growth of the K-562 cell line after 48 h treatment. (**a**) The CI values were calculated according to the Chou–Talalay method by CompuSyn software and plotted with the percent of cell growth inhibition as the fraction affected (Fa) cells. (**b**) Normalized isobologram for the combination. The symbols represent different combination ratios. Points that fall below the line are synergistic, above the line are antagonistic and on the line are additive.

The drug interaction between the hybrid compound and imatinib was studied using CompuSyn software. Drug synergism was determined with the help of the combination index (Figure 2a; Table 1) and isobologram (Figure 2b). Co-treatment of human leukemia cells with the hybrid and imatinib had a synergistic effect at all concentrations tested. However, treatment with 20 μM of the hybrid and 0.8 μM of imatinib gave the lowest CI value (0.27); as such, this combination of concentrations was chosen to continue the experiments.

Combination index values were generated by CompuSyn software using the formula CI = (D)_1_/(Dx)_1_ + (D)_2_/(Dx)_2_, where (Dx)_1_ or (Dx)_2_ represents the concentration of drug 1 or 2 in a combination needed for achieving the same efficiency as that of the single drug 1 or 2 at D1 or D2, respectively. CI < 1 indicate drug synergism, CI > 1 show antagonism and CI = 1 represent an additive effect.

### 2.3. The Hybrid Compound Induces Apoptosis by Increasing Caspase-3 and Caspase-9 Expression in K-562 and K-562R Cells

**Figure 3:**
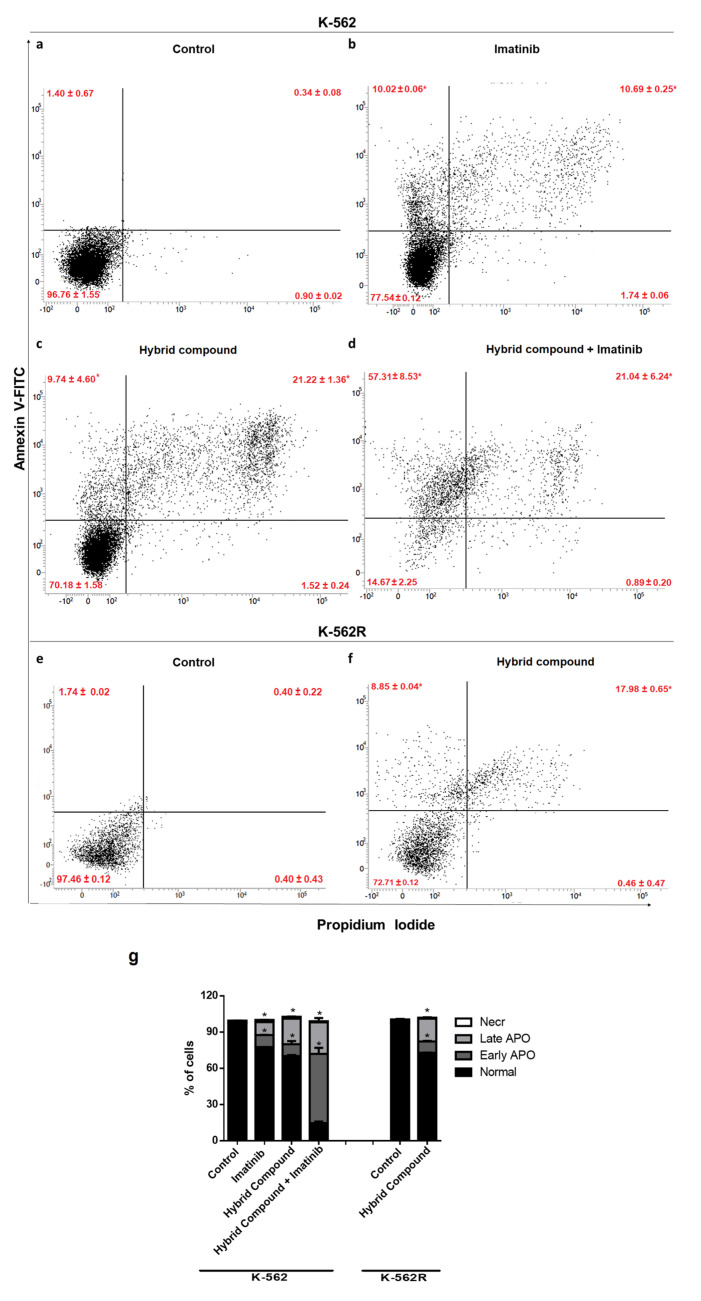
Representative analysis of apoptosis by flow cytometry after 48 h of treatment of K-562 cells with (**a**) only RPMI-1640 medium, (**b**) imatinib, (**c**) hybrid compound and (**d**) a combination of hybrid compound and imatinib, and of K-562R cells with (**e**) only RPMI 1640 medium or (**f**) hybrid compound. (**g**) Right, average values measured in each gate: NORMAL cells, annexin V−/IP−; NECR cells annexin, V−/IP+; EARLY APO cells, annexin V+/IP−; LATE APO cells and annexin V+/IP+. Data are reported as mean ± standard deviation (SD) of three experiments (* *p* < 0.05; relative to untreated control cells).

Flow cytometry showed that the incubation of K-562 and K-562R with the hybrid compound for 48 h aumented the quantity of early and late apoptotic cells (Figure 3). The apoptotic cell population significantly increased from 1.74% to 20.96% and from 2.01% to 26.83% in K-562 and K-562R cells, respectively (Figure 3c,f). Treatment with imatinib alone also increased the percentage of apoptotic cells from 1.74% to 20.72% (Figure 3b). Co-treatment led to an even greater increase in the labeling of apoptotic cells (from 1.74% to 78.35%; Figure 3d). 

**Figure 4:**
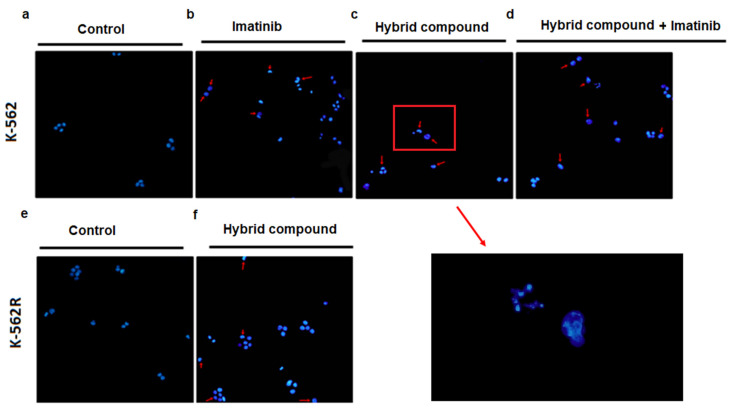
Apoptotic morphological changes observed by DAPI staining after 48 h of treatment of K-562 cells with (**a**) only RPMI-1640 medium, (**b**) imatinib, (**c**) hybrid compound and (**d**) a combination of hybrid compound and imatinib, and of K-562R cells with (**e**) only RPMI 1640 medium or (**f**) hybrid compound. Highlighted, the image allows the visualization of the apoptotic bodies. Classical apoptotic features such as cell shrinkage, blebbing and fragmented nuclei are highlighted (arrows).

To confirm whether apoptosis induction would alter cell morphology, DAPI staining was used. Figure 4 shows that all the treatments caused morphological changes in cells. These alterations were characterized by the formation of apoptotic bodies, condensation of the chromatin and the presence of fragmented nuclei with brighter blue fluorescence.

**Figure 5:**
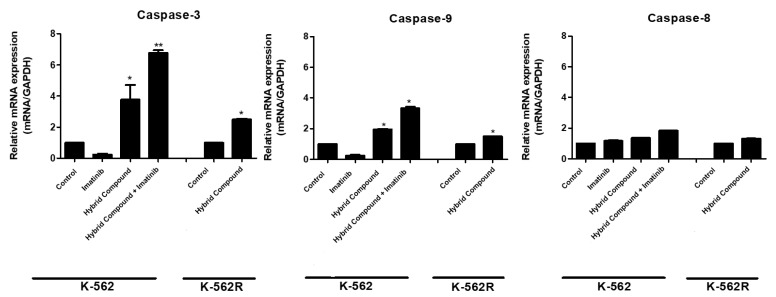
The mRNA expression levels of caspases 3, 9 and 8 were evaluated by qRT-PCR in K-562 cell line treated with imatinib, the hybrid compound and the combination of them, and in K-562R cells after treatment with the hybrid compound. Treatments were performed with respective IC_50_ for 48 h. Comparative analysis was performed with the 2^−ΔΔCT^ method using glyceraldehyde-3-phosphate dehydrogenase (GAPDH) as the endogenous control. The expression ratios were compared to the control. The values are representative of three different experiments. Data were compared by ANOVA followed by Tukey’s test (* *p* < 0.05 ** *p* < 0.01 represents statistical significance).

In addition, we determined whether the pathway by which apoptosis was triggered was intrinsic or extrinsic by evaluating the expression of caspases 3, 8 or 9 using qRT-PCR (Figure 5). Our results demonstrate that the hybrid treatment of K-562 and K-562R cells and the co-treatment of K-562 cells increases the expressions of caspase-3 and caspase-9, which are part of the intrinsic apoptosis pathway.

### 2.4. The Hybrid Compound Modulates the Cell Cycle Distributions of K-562 and K-562R Cells

**Figure 6:**
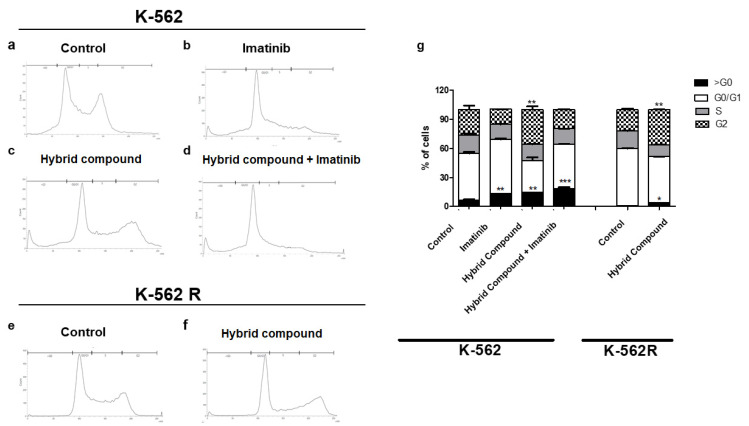
Cell cycle distribution after 48 h of treatment of K-562 cells with (**a**) only RPMI-1640 medium, (**b**) imatinib, (**c**) hybrid compound and (**d**) or combination of hybrid compound and imatinib, and of K-562R cells with (**e**) only RPMI 1640 medium or (**f**) hybrid compound (**g**) Percentage of sub-G0, G0/G1, S and G2 are shown. Data are reported as mean ± standard deviation (SD) of three experiments (* *p* < 0.05; ** *p* < 0.01; *** *p* < 0.001 relative to untreated control cells).

Data from the present study indicate that the hybrid may have induced cell cycle arrest in K-562 and K-562R cells. Flow cytometry was used to evaluate the cell cycle of K-562 and K-562R cells when treated with the hybrid (Figure 6). When we compared the hybrid-compound treated K-562 with the untreated control, we found a greater sub-G0 peak, characterizing cell death by apoptosis. The proportions of cells in the G2/M phase increased from 26.27% to 35.83% in K-562 cells and from 22.00% to 36.11% in K-562R cells (Figure 6c and Figure 6f, respectively). In contrast, the treatment of cells with imatinib affects the G0/G1 cell cycle arrest in K-562 cells (Figure 6b), where the proportion of cells in the sub-G0 and G0/G1 phase increased from 13.54% to 18.67% and from 32.95% to 45.75%, respectively, when comparing the hybrid compound treatment and co-treatment (Figure 6c and Figure 6d, respectively).

### 2.5. The Hybrid Compound Activates Autophagy by Increasing the Expressions of LC3-II and Beclin-1 in K-562 and K-562R Cells

**Figure 7:**
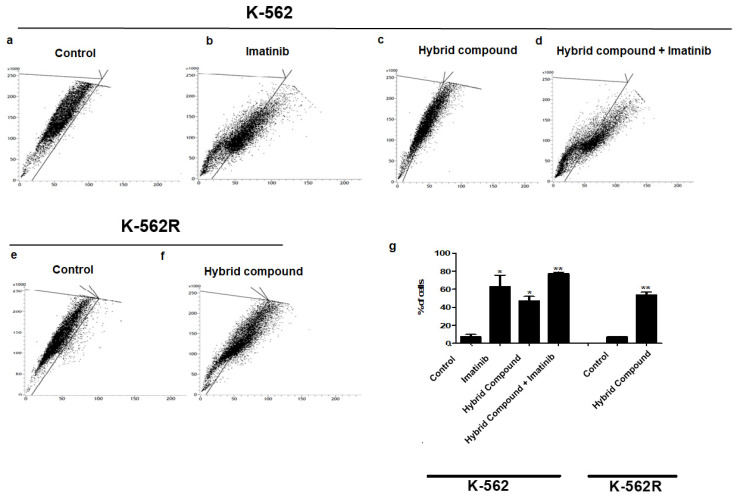
Autophagy observed with acridine orange (AO) staining by flow cytometry after 48 h of treatment of K-562 cells with (**a**) only RPMI-1640 medium, (**b**) imatinib, (**c**) hybrid compound and (**d**) a combination of hybrid compound and imatinib, and of K-562R cells with (**e**) only RPMI 1640 medium or (**f**) hybrid compound (**g**) Percentage of AO-positive cells are shown. Data are reported as mean ± standard deviation (SD) of three experiments (* *p* < 0.05; ** *p* < 0.01; relative to untreated control cells).

**Figure 8:**
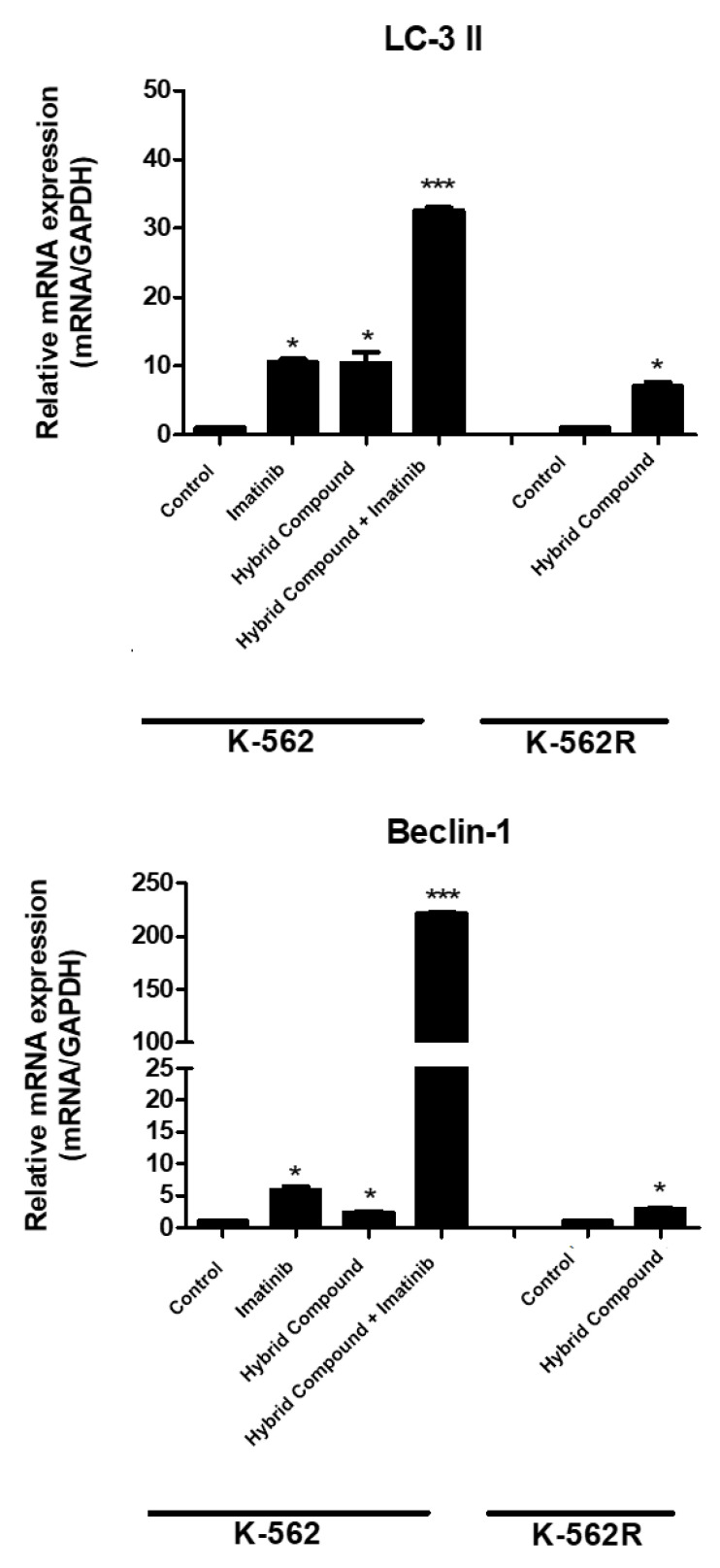
The mRNA expression levels of LC3-II and Beclin-1 were evaluated by qRT-PCR in K-562 cell line treated with imatinib, hybrid compound and the combination of them, and in K-562R cells after treatment with the hybrid compound. Treatments were performed with respective IC_50_ for 48 h. Comparative analysis was performed with the 2^−ΔΔCT^ method using glyceraldehyde-3-phosphate dehydrogenase (GAPDH) as the endogenous control. The expression ratios were compared to the control. The values are representative of three different experiments. Data were compared by ANOVA followed by Tukey’s test Data are reported as mean ± standard deviation (SD) of three experiments (* *p* < 0.05; *** *p* < 0.001 relative to untreated control cells).

To evaluate autophagy, acridine orange (AO) dye staining was used to indicate the maturation of autophagosomes. Both imatinib and the hybrid compound induced autophagy in K-562 cells (Figure 7b,c). The co-treatment resulted in 77.25% of the cells labeled with AO (Figure 7d). In K-562R cells, there was a significant increase of 53.95% in AO positive cells after treatment with the hybrid (Figure 7f). To confirm the autophagy mechanism induced by the hybrid compound, imatinib and co-treatment in the K-562 and K-562R cells, the gene expressions of two proteins that play key roles in the autophagic pathway were evaluated by qRT-PCR. In Figure 8, we show that all treatments increased the expression of autophagy markers, LC3-II and Beclin-1, in K-562 and K-562R cells, where the co-treatment of K-562 cells increased the gene expressions of these proteins by 32 ± 0.70 and 222 ± 1.41-fold, when compared to control cells.

### 2.6. The Hybrid Compound Induces Intracellular Reactive Oxygen Species (ROS) Generation in Human Leukemia Cells

**Figure 9:**
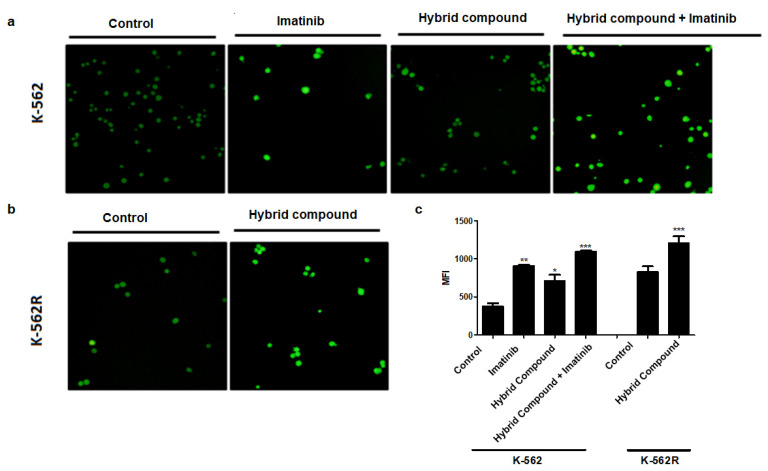
Influence of treatment with imatinib, hybrid compound and their combination on reactive oxygen species (ROS). Cells were stained with dihydroethidium (DHE), and ROS level was observed by fluorescence microscopy in (**a**) K-562 cell and (**b**) K-562R and (**c**) analyzed by flow cytometry. The values are representative of three different experiments. Data were compared by ANOVA followed by Tukey’s test Data are reported as mean ± standard deviation (SD) of three experiments (* *p* < 0.05; ** *p* < 0.01; *** *p* < 0.001 relative to untreated control cells).

To verify whether the hybrid compound generated ROS, cells were exposed to an oxidation-sensitive fluorescent dye, DCFH-DA, and cell fluorescence was determined using fluorescence microscopy and flow cytometry. Figure 9 shows that the hybrid, imatinib and co-treatment all induced ROS generation, where the co-treatment of K-562 cells induced the highest fluorescence (reported as medium intensity of fluorescence; MFI) (Figure 9b). The hybrid also increased the generation of ROS in K-562R cells (Figure 9c).

## 3. Discussion

The discovery of tyrosine kinase inhibitors, and the introduction of imatinib therapy in 2001, revolutionized the treatment of chronic myeloid leukemia [5]. However, the appearance of adverse effects elevated cases of treatment discontinuation. The main adverse reactions involving imatinib are gastrointestinal disturbances and rashes, in addition to thrombocytopenia and neutropenia [18,19,20]. Importantly, the discontinuation of treatment may lead to the development of therapeutic resistance; resistance to imatinib is a complex process without a defined cause. However, several studies demonstrate the participation of point mutations in the BCR-ABL protein, BCR-ABL overexpression and the expression of drug efflux pumps [21,22].

Natural compounds have been used in antineoplastic therapy not only as high performance and high-safety agents to induce cell death in leukemia cells, but also as a new therapeutic option in cases of resistance to classic chemotherapy, being used in combinations to try to avoid therapeutic resistance [4]. The main mechanism of action of chemotherapy is the induction of cell death via apoptosis; however, tumor cells may be resistant to this type of death [13]. As such, the search continues for new compounds capable of promoting multiple mechanisms of cell death, with maximum efficacy and few adverse effects.

In this study, we demonstrate the cytotoxic activity of a hybrid compound of betulinic acid (BA) and brosimine B on imatinib-sensitive (K-562) and imatinib-resistant (K-562R) cells. Even at low concentrations, the hybrid compound has a cytotoxic effect on K-562 cells; however, from concentrations of 25 µM the effects of the compound are similar in the two cell lines. In addition, the compound demonstrated a damage selectivity index that was 10-fold higher for tumor cells than for normal cells.

Synergism of this compound with imatinib was also observed in K-562 cells. The main advantage of this synergism is the possibility of a dose reduction to maintain or increase either agent’s therapeutic activity [14,23]. Our results demonstrate that low concentrations of imatinib and the hybrid compound have the same synergistic effect at high concentrations. This decrease in dose may lead to less therapeutic resistance and an improvement in treatment adherence, due to the decrease in the adverse effects associated with imatinib. Synergism with imatinib has already been reported with flavonoids in K-562 cells, where apigenin, luteolin and 5-desmethyl sinensetin have displayed synergic effects with imatinib [24].

The antitumoral activity and molecular mechanisms of BA have been reported in several studies [11]. Not many studies have investigated the mechanism of action of brosimine B in cancer, but a cytotoxic effect of this molecule has been shown in vincristine-sensitive and vincristine-resistant mouse leukemia P388 cells [7]. BA presents effects in combination with several chemotherapeutic drugs, such as etoposide, doxorubicin and cisplatin, to induce apoptosis [25]. A synergistic effect of BA with mithramycin A has also been observed in tumor pancreatic cells, where these molecules inhibit cell proliferation, invasion and angiogenesis [26].

Apoptosis is a mechanism of programmed cell death, characterized by morphological changes and by the formation of apoptotic bodies, chromatin condensation, nuclear fragmentation, microtubular alterations or mitotic defects [13]. Activation of apoptosis can be initiated by two different pathways, the extrinsic (cytoplasmic) or intrinsic (mitochondrial) pathways, in which the activities of different cysteine-dependent aspartate specific proteases (caspases) play an important role [27]. In the present study, the hybrid compound caused apoptosis in K-562 and K-562R cells, altering cell morphology and activating caspase-3 and caspase-9, which are part of the intrinsic pathway of apoptosis. Intrinsic apoptosis is mediated by mitochondrial outer membrane permeabilization, resulting in the activation of caspase-9, which directly activates caspase-3 and caspase-7 [13].

Our results corroborate findings in the literature indicating that BA promotes apoptosis in human hepatoblastoma, cervical and leukemia cell lines (HL-60 and K-562), altering the expressions of caspases of both the intrinsic and/or extrinsic pathways [28,29,30,31,32]. Treatment with flavonoids can also cause an increase in the activated forms of caspase 3, 8, and 9 and induce apoptosis in leukemia cells [33]. BA inhibits the PI3K/AKT cell-signaling pathway, which is an important antitumor target, especially for the induction of mitochondrial apoptosis. This signaling pathway is responsible for several cellular functions, including cell proliferation and tumor growth, and coordinates the cell cycle and cell migration, in addition to causing changes in the Bcl-2 and BAD family of proteins [13].

Furthermore, our findings demonstrate a significant pause at the sub-G0 phase of the K-562 and K-562R cell cycles, which also elicits death by apoptosis. Cell cycle arrest is crucial in the mechanism of action of some drugs, altering replication, proliferation and cell division, and has previously been described as one of the mechanisms of BA. Flavonoids and triterpenes induce the accumulation of leukemia cells in the G0/G1 and G2/M phases [33]. In the HeLa cell line, BA caused cell paresis in the G0/G1 phase, after inhibition of the PI3K/AKT pathway [28].

Another mechanism to activate apoptosis is via elevated ROS generation, since excess ROS can damage proteins and DNA, leading to cell death. Our study demonstrates that the hybrid compound induces the production of ROS, which could be a consequence of the activation of the caspase pathway and loss of the membrane potential [34]. In acute myeloid leukemia cells, BA combined with the histone deacetylase inhibitor increased ROS generation, in association with DNA damage, apoptosis and mitochondrial dysfunction [35]. Taxodice, another diterpene, induced apoptosis in K-562 cells and reduced the activities of mitochondrial respiratory chain complexes III and V, which appeared to induce the production of ROS [15]. There are several studies demonstrating the relationship between increased production of reactive oxygen species and anticancer studies. Yan Zhang and collaborators demonstrated that betulinic acid induces autophagy-dependent apoptosis via the Bmi-1/ROS/AMPK-mTOR-ULK1 axis in human bladder cancer cells, which corroborates our results [36]. In addition, Dash et al. demonstrated that nano-fibers of betulinic acid facilitated reactive oxygen species (ROS) mediated leukemic cell death, which was confirmed by pre-treatment of N-acetyl-L-cysteine. Induction of apoptosis by treatment increased pro-inflammatory cytokines, specifically potentiated TNF-α mediated cell death, which was confirmed by the expression of caspase-8 and caspase-3 by immunocytochemistry [37].

Autophagy is a physiological cellular process that leads to the degradation and recycling of damaged cellular components [13]. However, depending on the degree of activation, it can lead to cell death. The autophagic process can be monitored by the expression of related proteins such as LC3 I/II and Beclin-1. LC3 I is a cytosolic protein that is cleaved and converted to LC3 II after a pro-autophagic stimulus. Beclin-1 is part of a type III phosphatidylinositol 3-kinase complex that is required for autophagic vesicle formation. In cancer, autophagy exhibits a contradictory behavior and, depending on the cell type, it may be an important factor for the induction of cell death or tumor progression [38]. It is now known that imatinib induces autophagy in the K-562 cell line and in primary cultures of patients with CML, although this induction was not associated with BCR-ABL activity, but rather with endoplasmic reticulum stress [39,40]. Our results show that the hybrid compound causes increased autophagy, as demonstrated by acridine orange staining in the K-562 and K-562R cell lines, in addition to increasing the expressions of LC3II and Beclin-1. Chemotherapies also cause autophagy, as is the case of 5-fluorouracil in HCT116 human colon cancer cells [41]. Other studies also report the induction of autophagy by BA via the inhibition of the AKT-mTOR signaling pathway [42]. The alkylated derivative of quercetin, 7-O-geranylkercitin, induced autophagosome formation in A549 and NCI-H1975 cells, promoting the expression of the autophagy-related proteins, LC3-II and Beclin-1, and increased ROS generation [42]. The search continues for molecules capable of presenting different mechanisms to avoid therapeutic resistance; however, there is still no consensus as to which proteins link the cell death pathways [42,43]. Although our study does not contain Western blot data, our results corroborate with Ghiubai et al., who found that the betulic acid nanoformulation induces selective cytotoxic ativity. Examinations revealed a pro-apoptotic effect, as evidenced by morphological changes in melanoma cells and supported by Western blot data showing the downregulation of anti-apoptotic Bcl-2 expression coupled with the upregulation of pro-apoptotic Bax [44].

In conclusion, our results demonstrated that the hybrid compound induces a number of molecular mechanisms in K-562 and K-562R cells, inducing apoptosis via caspases 3 and 9, cell cycle arrest and autophagy death with the increased expression of LC3II and Beclin-1. Our hybrid combination with imatinib had lower doses with the same synergistic effect. Consequently, we could have a reduction in resistance to chemotherapy and greater adherence by patients due to the reduction of adverse effects. These results are promising and protein Western blotting studies, as well as in vivo studies with animals and with patient samples, are needed to confirm the possibility of the development of new anticancer treatments against CML.

## 4. Materials and Methods

### 4.1. Cell Culture

The chronic myeloid leukemia cell line (K-562) was purchased from the Rio de Janeiro cell bank (Banco de Células do Rio de Janeiro (BCRJ), Rio de Janeiro, Brazil). Cells were cultured in RPMI-1640 medium with 10% fetal bovine serum and 100 U/mL pen-streptomycin and incubated at 37 °C in 5% CO_2_. The K-562R (imatinib-resistant cell line) was generated as described by Willig et al. [18]. Peripheral blood mononuclear cells (PBMNCs) were used as control and were obtained by centrifuging blood from healthy donors over a Histopaque®-1077 (Sigma-Aldrich, St. Louis, MO, USA) density gradient. This project was approved by the Ethics Committee of the Federal University of Rio Grande do Sul (n.1.979.570). All donors signed informed consent.

### 4.2. Synthesis of Hybrid Compound

**Figure 10:**
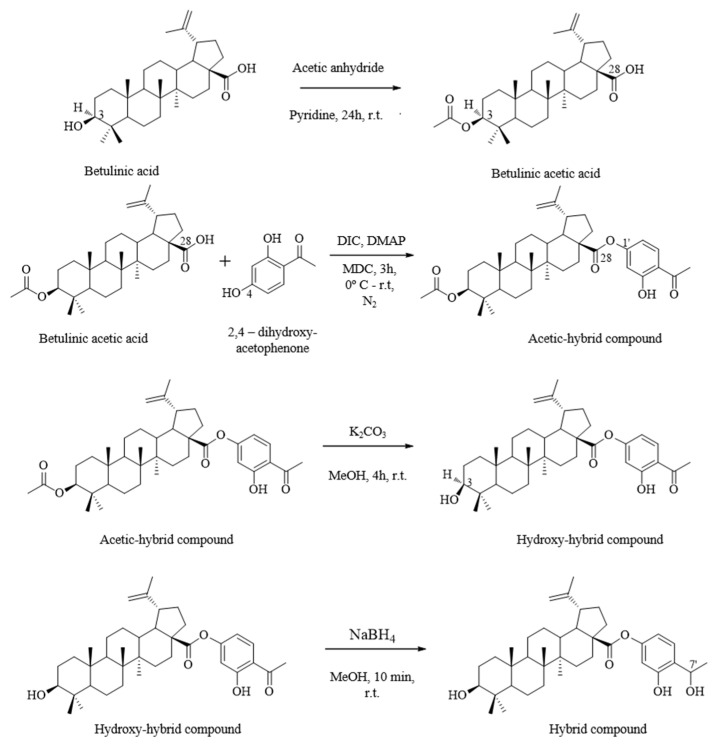
Illustrative scheme of hybrids synthesis from their natural precursors.

The triterpene-flavonoid hybrid compound was synthetized as described by Couto, et al. [12] and shown in Figure 10. The synthesis is carried out from the natural product (betulinic acid), which is widely available and involves low-complexity steps.

### 4.3. Cell Counting

Cells were seeded onto 96-well microplates (8 × 10^3^/well) and then incubated at 37 °C in 5% CO_2_ for 24 h. Afterwards, cells were treated with the hybrid compound (0–75 µM) for another 48 h. Cell counting was performed in a FACSVerse™ cytometer equipped with a 488 nm blue laser and flow sensor (BD Biosciences, San Jose, CA, EUA). Results are expressed as the percentage of the control. Dose-response curves were constructed and IC_50_ values were determined by GraphPad Prism software (version 8.0). The IC50 of imatinib was previously described by Waechter F. et al. [10].

### 4.4. Selectivity Index (SI) Analysis

PBMNCs were treated to assess the selectivity index (SI) in order to evaluate how selective the hybrid compound is for killing/damaging cancer cells instead of normal cells. The degree of selectivity of the hybrid compound was expressed for each tumor cell line, according to the equation SI = CC_50_/IC_50_ [10]_._

### 4.5. Analysis of the Effects of Drug Combinations

K-562 cells were seeded onto 96-well microplates (8 × 10^3^/well) for 24 h before exposing them to different concentrations of the hybrid compound (5, 10, 20, 40, 80 µM), imatinib (0.2, 0.4, 0.8, 1.6, 3.2 µM) or their combination. After 48 h of treatment, cells were counted with a FACSVerse™ cytometer. CompuSyn software was used to calculate the combination index (CI) and isobologram to quantitatively determine the effect of drug interactions. CI values of less than 1, equal to 1 and greater than 1 represent synergism, additivity and antagonism, respectively. The isobologram is formed by plotting the concentrations of each drug for 50% inhibition (EC50) on the X- and Y-axis and connecting them with a line segment, which is the ED50 isobologram. Combination points that fall on, below and above the line segment represent additivity, synergism and antagonism, respectively [14].

### 4.6. Evaluation of Cell Death

For evaluation of the mechanism of cell death, K-562 and K-562R cells were treated with the concentration of the corresponding IC_50_ of the hybrid compound for 48 h. K-562 cells were also treated with imatinib and a combination of imatinib and the hybrid compound for 48 h.

#### 4.6.1. Annexin V–FITC/PI Staining

Phosphatidylserine (PS) externalization was determined by the annexin fluorescence signal of an annexin V–fluorescein isothiocyanate conjugate (Quatro G Pesquisa & Desenvolvimento, Porto Alegre, Brazil), according to the manufacturer’s protocol. Briefly, cells were treated at the corresponding concentrations for 48 h, then harvested, centrifuged for 5 min at 1500 rpm and the supernatants were discarded. Pellets obtained were re-suspended with 150 μL of annexin binding buffer. Cells were stained with 5 μL annexin V and 5 μL propidium iodide for 15 min at room temperature in the dark. The apoptotic index was immediately determined on a FACSVerse™ flow cytometer (BD Biosciences).

#### 4.6.2. Detection of Morphological Changes

To detect morphological changes that occurred during the apoptosis process, nuclear staining was performed using DAPI. After 48 h of treatment, cells were harvested and fixed in 4% paraformaldehyde for 20 min. Subsequently, cells were stained with 4 µg/mL DAPI (Sigma, St Louis, MO, USA) for 30 min at room temperature. After washing with PBS, samples were stored in the dark at 4 °C and visualized on a fluorescence microscope (Olympus IX71 microscopy).

#### 4.6.3. Cell Cycle Analysis

Cell lines were seeded onto 6-well plates (1 × 10^6^ cells/well) and incubated for 24 h at 37 °C in a humidified incubator, as previously described. Cells were treated using the corresponding concentrations of treatment and control cells were maintained with just RPMI 10% PBS. After a 48-h period, cells were harvested and fixed in 70% ice-cold ethanol (*v*/*v* in PBS) for 24 h. Then, cells were then washed with PBS and a solution containing 12 μg/mL propidium iodide, 0.1% Triton X-100 and 50 μg/mL RNAse was added to each tube [19]. After 30 min of incubation at room temperature, protected from light, cells were analyzed using a FACSVerse™ flow cytometer (BD Biosciences).

#### 4.6.4. Autophagy Analysis

Autophagy analysis was performed in 24-well plates, where cells were seeded (30 × 10^4^ cells per well) and maintained for 24 h at 37 °C and 5% CO_2_. Afterwards, cells were treated for 48 h. Posteriorly, cells were incubated with acridine orange (AO) (2.7 μM) for 15 min at room temperature, as described previously. Cell staining was determined using the FACS Verse flow cytometer (BD Biosciences, San Jose, CA, USA).

### 4.7. Quantitative Reverse Transcription-Polymerase Chain Reaction (RT-qPCR)

Total RNA was isolated from cells using the TRIzol reagent method (Life Technologies, Carlsbad, CA, USA) and quantified with a Nano-Drop^®^ spectrophotometer (Thermo Scientific, Waltham, MA, USA). cDNA was synthesized from 5 µg of total RNA in total volume reactions (20 µL) with M-MLV reverse transcriptase (Life Technologies, Carlsbad, CA, USA). Real-time polymerase chain reactions were performed in a Rotor-Gene Q (Qiagen, Germantown, MD, USA) using 1 µL cDNA, specific primers (Table 2) and GoTaq^®^ qPCR Master Mix (Promega, Madison, WI, USA), according to the manufacturer’s instructions. The conditions applied were 95 °C for 1 min, 40 cycles of 10 s at 95 °C, 15 s at 60 °C and 20 s at 72 °C. The relative expression levels of the genes were determined by the 2^−ΔΔCT^ method, and glyceraldehyde-3-phosphate dehydrogenase (*GAPDH)* was used as an internal control.

### 4.8. Determination of ROS Generation

Dihydroethidium (DHE) was used to detect reactive oxygen species (ROS) generation. After treatment, cells were incubated with 10 μM DHE for 30 min. Fluorescence was observed using a fluorescence microscope (Olympus IX71 microscopy) and a FACSVerse flow cytometer (BD Biosciences, San Jose, CA, USA).

### 4.9. Statistical Analysis

Experiments were performed three times (*n* = 3) with samples in triplicate, and the results were analyzed by analysis of variance (ANOVA), followed by the Tukey test. The software used was GraphPad Prism (version 8.0). Significant values were considered for *p* < 0.05.

## Figures and Tables

**Table 1:** Hybrid compound and imatinib concentrations used in the different combinations evaluated and their respective combination index.

Drugs Combinations		Combination Index (CI)
Hybrid compound (µM)	Imatinib (µM)	
5	0.20	0.59
10	0.40	0.37
20	0.80	0.27
40	1.60	0.44
80	3.20	0.38

**Table 2:** Sequence, annealing temperature and product size of the primers used for qRT-PCR experiments.

Gene Product	Primer Sequences	Annealing Temperature	Product Size (bp)
*Caspase-3*	F-5′-ACATGGCGTGTCATAAAA-3′ R-5′-CACAAAGCGACTGGAC-3′	60 °C	120
*Caspase-8*	F-5′-CTGCTGGGGATGGCCACTGTG-3′ R-5′-TCGCCTCGAGGACATCGCTC-3′	60 °C	113
*Caspase-9*	F-5′-GAGTCAGGCTCTTCCTTTG-3′ R-5′-CCTCAA ACTCTCAAGAGCAC-3′	60 °C	241
*LC3-II*	F-5′-GAGAAGCAGCTTCCTGTTCTGG-3′ R-5′-GTGTCCGTTCACCAACAGGAAG-3′	60 °C	138
*Beclin-1*	F-5′-GGCTGAGAGACTGGATCAGG-3′ R-5′-CTGCGTCTGGGCATAACG-3′	60 °C	127
* GAPDH *	F: CAAAGTTGTCATGGATGACC R: CCATGGAGAAGGCTGGGG	60 °C	195

## Data Availability

All data are available in the article or by contacting the corresponding author.
